# Temporal Exposure to Bt Insecticide Causes Oxidative Stress in Larval Midgut Tissue

**DOI:** 10.3390/toxins15050323

**Published:** 2023-05-07

**Authors:** Biko K. Muita, Simon W. Baxter

**Affiliations:** 1 School of Biological Sciences, University of Adelaide, Adelaide 5005, Australia; biko.muita@adelaide.edu.au; 2 School of BioSciences, University of Melbourne, Melbourne 3010, Australia

**Keywords:** Bt toxin, Cry1Ac, ABCC2, transmission electron microscopy, RNA-seq

## Abstract

*Bacillus thuringiensis* (Bt) three-domain Cry toxins are highly successful biological pesticides; however, the mechanism through which they cause death to targeted larval midgut cells is not fully understood. Herein, we challenged transgenic Bt-susceptible *Drosophila melanogaster* larvae with moderate doses of activated Cry1Ac toxin and assessed the midgut tissues after one, three, and five hours using transmission electron microscopy and transcriptome sequencing. Larvae treated with Cry1Ac showed dramatic changes to their midgut morphology, including shortened microvilli, enlarged vacuoles, thickened peritrophic membranes, and swelling of the basal labyrinth, suggesting water influx. Transcriptome analysis showed that innate immune responses were repressed, genes involved with cell death pathways were largely unchanged, and mitochondria-related genes were strongly upregulated following toxin exposure. Defective mitochondria produced after toxin exposure were likely to contribute to significant levels of oxidative stress, which represent a common physiological response to a range of toxic chemicals. Significant reductions in both mitochondrial aconitase activity and ATP levels in the midgut tissue supported a rapid increase in reactive oxygen species (ROS) following exposure to Cry1Ac. Overall, these findings support the role of water influx, midgut cell swelling, and ROS activity in response to moderate concentrations of Cry1Ac.

## 1. Introduction

The digestive tracts of insects are normally exposed to a variety of bacteria that are ingested during feeding. Although some bacteria are beneficial, larvae must eliminate virulent species and recover from any toxic factors they produce, such as proteases and peptides. The basic structure of the gut is relatively conserved in most insect species, despite a broad spectrum of diets [1]. Generally, the insect gut is organized into three main sections: the foregut, which includes the pharynx, esophagus, and crop, a structure used to store food; the midgut, which carries out food digestion and nutrient absorption; and the hindgut, the major site of water absorption [2]. The midgut is made up of a single layer of epithelial cells on a basement membrane of connective tissue surrounded by a thin layer of muscle [3]. The intestinal epithelium plays a role in defining the barrier between the host and the external environment, enabling control over the invasion and systemic dissemination of both pathogenic and commensal microorganisms within the body cavity [4,5]. 

The gram-positive pathogen *Bacillus thuringiensis* (Bt) produces insecticidal proteins, including three-domain Cry toxins, that disrupt the midgut epithelial layer to allow for bacterial invasion. Crystalline Bt toxins are produced during bacterial sporulation, and, following ingestion, are solubilized into 70–130 kDa protoxins in alkaline insect gut environments. Subsequent processing by proteases activates the ~60–67 kDa toxin, which then binds to specific receptors on the surface of the midgut brush border membrane. Bt has been an important biological pesticide product for over 50 years, as it is generally active on a narrow spectrum of insect targets [6], and hundreds of protein toxin varieties have been described [7]. Bt products are biodegradable and used in both organic and conventional farming as foliar sprays [8]. Genes encoding insecticidal toxin proteins have been expressed in a range of transgenic Bt crops and used successfully to control a variety of insect pests [9], although insecticidal resistance to Bt products can occur [10,11]. 

Multiple receptor classes for Cry1A toxins have been identified from a broad range of Lepidoptera species. These include an aminopeptidase-N (APN) and Cadherin-like protein (CaLP), which were identified in *Manduca sexta* [12,13]; and alkaline phosphatase (ALP)- and ATP-dependent binding cassette transporter C2 (ABCC2), first identified in *Heliothis virescens* [14,15]. Other potential receptors and proteins associated with Bt resistance have been described [16]. Knock-out of an ABCC2 paralogue, ABCC3, in *Plutella xylostella* was found to confer a >500-fold resistance to Cry1A toxin [17], although subsequent studies showed that a double knock-out of ABCC2 and ABCC3 was required to confer a high level of resistance [18]. ABC transporters have been proposed as the most important receptor class required for Bt pore formation [19], and can act synergistically with other receptor types [20,21,22].

Despite being extensively studied, aspects of Bt toxin’s mode of action remain unresolved and may vary between species. Several models have been proposed to explain how Bt toxins interact with midgut receptors to form pores, which leads to larval death. The sequential binding model proposes that the toxin monomer binds to CaLP, which cleaves an N-terminal α1-helix subunit whereupon oligomeric pre-pores are formed. The pre-pores then bind to a second receptor, such as ABCC2, then insert into the membrane to form cation-selective pores [23]. Cell death is believed to be a result of cell lysis due to the formation of toxin pores [24,25,26,27]. Binding of the monomer to CaLP, however, is not necessarily a requirement for toxicity, as *Helicoverpa armigera* CaLP null-mutants remain susceptible to high toxin doses [28]. Expressing an ABCC2 transgene from *P. xylostella* in *Drosophila melanogaster* larval midguts confers susceptibility to the Cry1Ac toxin, despite *D. melanogaster* lacking a CaLP orthologue [29]. A separate Bt insecticidal mode of action model suggests that binding of Bt toxin to CaLP causes cytotoxicity through activation of a Mg^2+^ dependent adenylyl cyclase/PKA signaling pathway, leading to cell death [30]. 

*Plutella xylostella*, the diamondback moth, is a worldwide pest of brassica crops such as canola and broccoli, with control costs and crop yield losses predicted at around USD 5 billion annually [31]. It was the first pest to develop field resistance to the Cry1Ac toxin due to a 10-amino-acid deletion in toxin receptor ABCC2 [32]. Stevens et al. [29] transformed *P. xylostella ABCC2* (PxABCC2) into *D. melanogaster*, then expressed the protein in larval midgut tissue using the GAL4-UAS system. *Drosophila melanogaster* is not a pest or susceptible to activated Bt Cry1Ac toxins; however, expressing PxABCC2 in the midguts of *D. melanogaster* larvae was sufficient to confer susceptibility with an LC_50_ concentration of 78 µM. This in vivo model system provides a useful resource for developing a better understanding of the modes of action of Bt toxins. Here, we performed transcriptional analyses and temporal observations using transmission electron microscopy to document the response to moderate concentrations of Cry1Ac in *D. melanogaster* larval midgut tissue expressing the *P. xylostella* ABCC2 transgene. Elevated levels of reactive oxygen species (ROS) were observed in midgut tissue treated with Cry1Ac, which is consistent with the stress-induced responses produced by a range of chemical insecticides. 

## 2. Results

### 2.1. Cry1Ac Damages D. melanogaster Larval Midgut Epithelial Cells Expressing PxABCC2

The concentration of activated Cry1Ac toxin required to kill 50% of transgenic *D. melanogaster* expressing PxABCC2 during development was previously determined (LC_50_ concentration of 78 µM) [29]. Here, we treated third instar larvae using this benchmark concentration of Cry1Ac over short time periods to document the progressive impact that moderate toxin doses have on midgut tissue. Third instar larvae were observed feed-actively on artificial diet containing Cry1Ac for the first hour, and generally avoided the food source after three hours. Cry1Ac feeding assays were, therefore, performed on 3rd instar larvae over a limited time series of 1 h, 3 h, and 5-h to capture active feeding and avoidance phases. Control midgut regions from untreated reference groups were fed on a standard diet for 1-h. Anterior midgut regions were dissected from a minimum of three biological replicates for each group and visualized using transmission electron microscopy (TEM). Representative images of transverse sections of larval midguts were obtained using multiple magnification settings to record changes to cell structures over time. The physical effects of the toxin on morphology were identified by comparing the images of Cry1Ac toxin-treated midgut samples with untreated controls.

**Figure 1:**
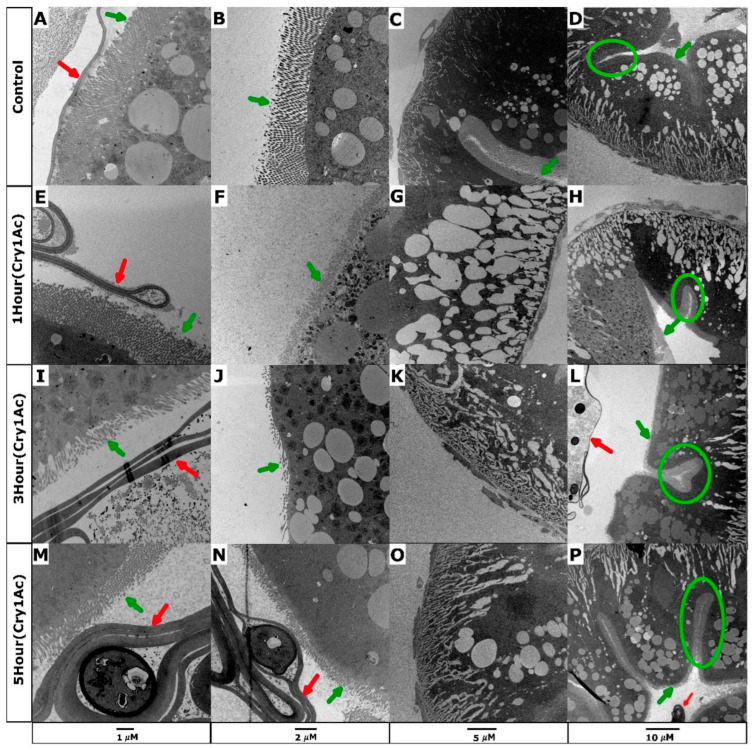
Transmission electron microscopy images highlight changes to the morphology of *D. melanogaster* larval midgut sections in response to moderate concentrations of Cry1Ac. Imaging shows *D. melanogaster* GAL4-NP1/UAS-PxABCC2 midgut regions near the crop with 1 µm, 2 µm, 5 µm and 10 µm scale bars. Colored arrows indicate microvilli (green) and the peritrophic membrane (red). (**A**–**D**) Control larvae show epithelial cells with long microvilli and a narrow peritrophic membrane. (**E**–**H**) Samples treated for 1 h show short, disorganized microvilli, swollen vacuoles, and dilated basal labyrinths. (**I**–**L**) Samples treated for 3 h show shortened or sheared microvilli, enlarged vacuoles, and swollen basal labyrinths. (**M**–**P**) The samples treated for 5 h show microvilli with increased lengths relative to the 3 h timepoint, which may represent a degree of recovery. Moderate basal labyrinth swelling and large numbers of vacuoles are evident.

**Figure 2:**
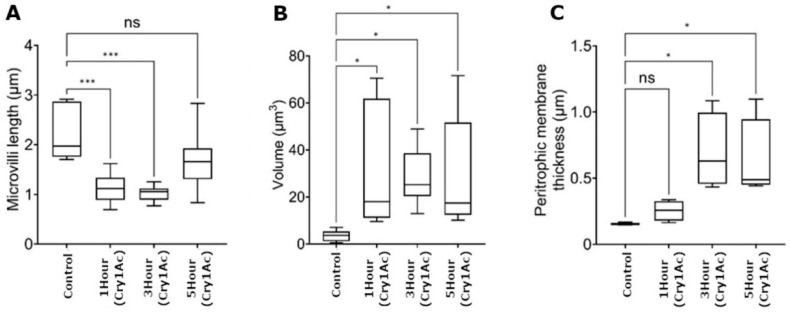
Quantitative assessment of larval midgut tissues following 1, 3 or 5 h of exposure to moderate doses of Cry1Ac. (**A**) Transmission electron microscopy images show that microvilli were significantly shortened following 1 and 3 h of Cry1Ac treatment. Following 5 h, several sections of microvilli were similar in length to the control. (**B**) Vacuole volume significantly increased, but was highly variable, among the image sections. (**C**) Peritrophic membrane thickness increased considerably after 3 and 5 h. ImageJ was used to record measurements from ~14 TEM images taken from 3 biological replicates per treatment group. Statistical significance was determined by one-way ANOVA and Dunnett’s multiple comparisons test (* *p* < 0.05, *** *p* < 0.001).

Microvilli function to increase the surface area of the brush border membrane and facilitate nutrient absorption. High-resolution images of control larval midgut sections displayed tightly packed microvilli that appeared long and thin (Figure 1A,B). Following 1 h of toxin exposure, damage was evident on the apical membrane, where microvilli appeared to thicken and shorten in length (Figure 1E,F). The damage progressively increased at 3 h, where the microvilli had largely sheared (Figure 1I,J). Contrary to expectations of continued progressive shortening, the microvilli appeared to undergo a level of regeneration after 5 h and regain structures resembling control tissue, despite Cry1Ac remaining accessible to larvae (Figure 1M,N). Invaginations within the brush border membrane appeared, creating small pockets of microvilli that were similar in structure to the controls, suggesting that the Cry1Ac toxin was ineffective in these regions (Figure 1D,H,L,P green circle). Measurement of the microvilli confirmed a significant reduction in length from ~2 µm in the controls to ~1 µm after 1 h and 3 h (Figure 2A). The lengths of the microvilli were ~1.8 µm after 5 h, which was relatively consistent with the control tissue. Regeneration or repair of microvilli may occur during periods of physical toxin avoidance, thus minimizing the uptake of additional insecticidal protein.

**Figure 3:**
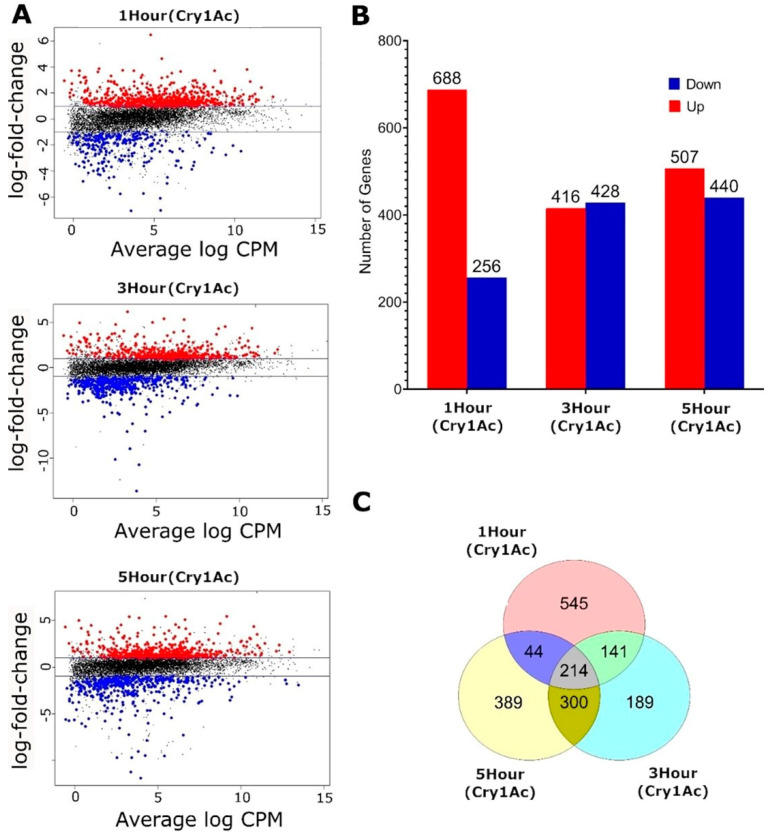
Exposure to Cry1Ac toxin in *D. melanogaster* (NP1−GAL4;UAS−PxABCC2) larvae influences transcription over time. (**A**) Mean-difference (MD) plots showing the log-fold change and average abundance of each gene relative to the control group. Genes with log-fold changes greater than 1 and FDR < 0.05 are highlighted. (**B**) Bar graph showing the number of genes with increased and reduced expression at each timepoint. (**C**) Venn diagram of differentially expressed genes between treatments when compared to the same control.

Midgut sections that were exposed to the toxin showed considerable changes in the organization of cellular structures relative to the control. The dark, circular, organelle-like structures were observed after 1 h (Figure 1F) or 3 h (Figure 1I,J) Cry1Ac treatments are potentially mitochondria. Increases in the density and size of vacuole were observed after 1 h of exposure, which demonstrated that vacuoles emanated from the basal labyrinth (Figure 1G,H). Similar levels of swelling and vacuolization were also visible after 3 and 5 h (Figure 1K,L,O,P). The quantification of the total vacuole volume using three biological replicates confirmed a significant increase in all treatment groups (*p* < 0.05), although high levels of variation did occur between images (Figure 2B). Expansive basal labyrinth regions were found near the basal membrane in all images captured at 5–10 µm. Cry1Ac-treated samples were generally dilated, particularly after the 1 h treatment (Figure 1G,H). 

The peritrophic membrane forms a barrier-like structure between the lumen and gut epithelial cells and plays a protective role against infection by bacterial pathogens [33]. Thickening of the peritrophic membrane occurred in response to Cry1Ac, increasing the membrane width by 3-fold (~0.5 µm) over 3 h (Figure 2C). Transmission electron microscopy highlighted a clear morphological response to moderate doses of Cry1Ac, but did not confirm cell lysis or indicate evidence of cell death at this experimental concentration.

### 2.2. Transcriptomic Responses to Cry1Ac in the D. melanogaster Larval Midgut

Transgenic *D. melanogaster* third instar larvae were fed on either a standard diet for 1 h or diet containing activated Cry1Ac (78 μM) for 1, 3, or 5 h. Midguts were dissected, and total RNA was isolated from the crop and apical midgut. Three biological replicates, each containing eight midguts, were produced for these four groups, generating twelve libraries for RNA sequencing. After quality filtering, each library contained an average of 39.3 ± 9.2 million high-quality reads. About 87.9% ± 7% of the *D. melanogaster* reads were mapped to the *D. melanogaster* (BDGP6) reference genome (Table S1).

A total of 17,738 transcripts were identified, and genes with low read counts (<1 CPM in at least 3 samples) were excluded, leaving 8132 transcripts for analysis. Differential expression analysis was carried out by comparing each treatment group to the control values. Differentially expressed genes were identified using an adjusted false discovery rate (FDR) with a *p*-value < 0.05 and a log_2_ ratio > 1.2 (Figure 3A). Samples treated with Cry1Ac for 1 h had 688 genes with increased expression and 256 genes with reduced expression relative to the untreated control. Treatments for 3 h showed 416 genes with increased expression and 428 genes with reduced expression, while 5 h treatments showed 507 and 440 genes with increased and reduced expression, respectively (Figure 3B). All 3 treatment groups shared 214 common differentially expressed genes (103 increased and 111 reduced) (Figure 3C).

Antimicrobial peptides are involved in the immune response to bacteria. Expression patterns varied in response to Cry1Ac, with some reductions observed in *Dpt A* and *CG43236*, as well as increased expression of *AttD*, *CecA1*, *CecA2* and *AttB*. Multiple lysosomal and IMD pathway genes [34] that may have roles in the defense mechanism against *Bacillus* were also differentially expressed (Table 1).

The expression of autophagy and apoptotic cell death pathway genes showed variable responses to Cry1Ac (Table 2). The initiator caspase *Dronc* increased in expression to all treatments, and *Dcp-1*, an effector caspase, increased expression following 3 and 5 h of Cry1Ac exposure. However, HiD, an activator of apoptosis, p53, and myc, inducers of apoptosis through the transactivation of target genes, had reduced expression with 1 h of treatment. Autophagy-related genes essential for activation, such as Atg13a and Atg18a, were not significantly expressed, suggesting that autophagy is not activated under these experimental conditions.

Differentially expressed genes were analyzed using GO and KEGG to identify enriched terms and pathways among the treatment groups. Upregulation of mitochondrial-related and oxidation–reduction processes were identified in the 1 h treatment group, and, to a lesser degree, in the 3 h treatment group (Tables S2 and S3). The 5 h treatment group displayed downregulation of terms related to the nucleus and RNA processing. Signaling pathways JNK, JAK/Stat, Hippo, EGFR, Wg, Hh, and Dpp/BMP, as well as autophagy- and apoptosis-related terms were not enriched in any of the treatment groups. 

**Figure 4:**
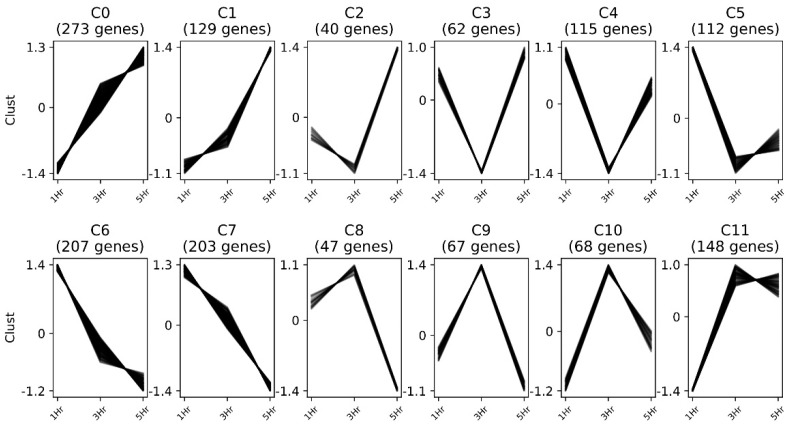
Clustering analysis of 1471 differentially expressed genes in response to Cry1Ac treatments for one, three or five hours (1 h, 3 h, 5 h). Differentially expressed genes were clustered into twelve groups that produced four general profiles. *Y*-axis values are normalized z-scores.

Clust [35] partitioned genes showing significant levels of genetic differentiation (n = 1471) into 12 cluster groups, which were defined by their expression profiles (Figure 4, Table S4). The clusters highlighted four predominant trends: (i) increased expression over time (C0–C1); (ii) genes with reduced expression at 3 h (C2–C5); (iii) genes with initially elevated expression that reduced over time (C6–C7); and (iv) genes with higher expression among the 3 h treatment group (C8–C11). These data suggest a diverse and dynamic transcriptomic response to toxin exposure that is affected by the duration of exposure. 

**Figure 5:**
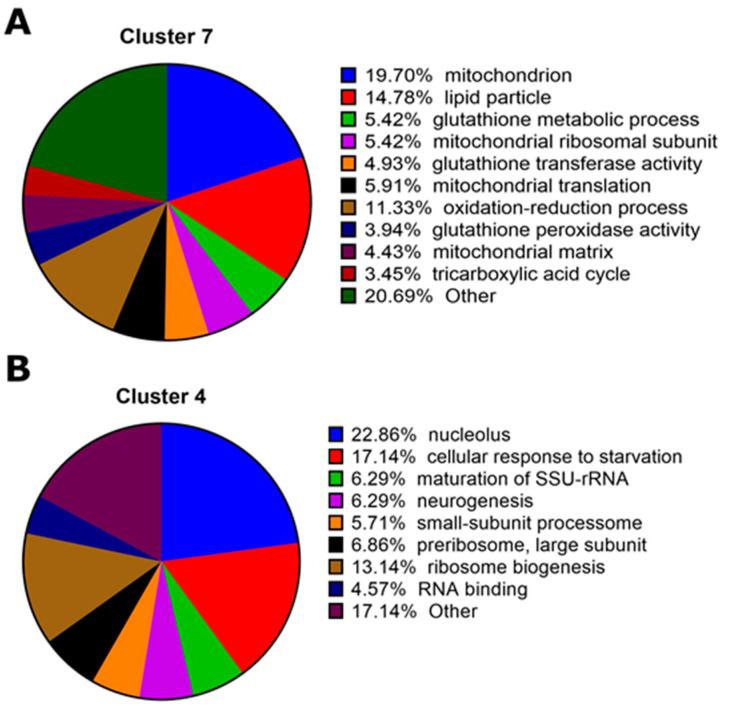
Gene ontology (GO) analysis of two prominent gene expression clusters over time (C4 and C7, see Figure 4 for cluster profiles). (**A**) Gene expression of C7 was elevated after *D. melanogaster* larvae fed on Cry1Ac for 1 h; it primarily involved factors involved with mitochondrion energy production in response to cellular stress and damage. (**B**) Cluster C4 grouped genes with decreased expression levels only after 3 h of exposure to Cry1Ac, and included factors associated with the response to starvation, suggesting that insufficient nutrition was available to maintain cellular functions. Genes involved with ribosome production (e.g., nucleolus, pre-ribosome large subunit, ribosome biogenesis) were also identified.

A gene ontology (GO) analysis on each of the twelve clusters was performed using DAVID GO [36] to identify the biological functions that were enriched in each group (Tables S5–S7). Clusters C4 and C7 had the largest number of enriched terms (Table S4). Cluster C7 showed a progressive reduction in gene expression over time, with enriched cellular component terms including “mitochondrion” and “lipid particle” as well as biological processes terms “oxidation-reduction process” and “mitochondrial translation” (Figure 5A). Cluster C4 showed slightly increased expression in the first hour which reduced at three hours and increased at five hours. GO enrichment highlighted the biological processes involved in the “cellular response to starvation” in line with changes in behavior to cease feeding after toxin ingestion. They were also enriched for the cellular component “nucleolus” (Figure 5B).

### 2.3. qPCR Validation of 10 D. melanogaster Genes

**Figure 6:**
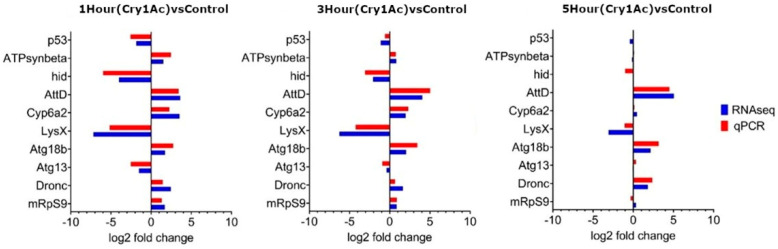
Comparison of gene expression levels from *D. melanogaster* larval midgut tissue, determined with RNA-seq transcriptomics (blue) or quantitative reverse transcription PCR (red). Ten genes identified with significant differential expression within RNA−seq datasets were re-analyzed using qPCR. mRNA levels estimated using qPCR were normalized with housekeeping gene Rp49. Three biological replicates and three technical replicates were analyzed for one hour (left image), three hours (center), and 5 hours (right) of Bt Cry1Ac treatment versus an untreated control. All assessed genes showed similar qPCR and RNA−seq expression profiles at each timepoint. The FlyBase gene abbreviation and number included p53, FBgn0039044; ATPsynbeta, FBgn0010217; hid, FBgn0003997; AttD, FBgn0038530; Cyp6a2, FBgn0000473; LysX, FBgn0004431; Atg18b, FBgn0032935; Atg13, FBgn0261108; Dronc, FBgn0026404; and mRpS9, FBgn0037529.

Quantitative PCR was performed on ten genes that showed significant differential expression in RNA-seq data in order to determine whether consistent expression values could be obtained. Genes were selected due to their involvement in antimicrobial activity; mitochondrial production; or ATP synthesis, autophagy, and apoptosis. RNA isolated for quantitative PCR and RNA-seq was generated from independent biological assays. All genes analyzed using qPCR showed similar expression profiles to that of the RNA-seq data at each timepoint (Figure 6). 

### 2.4. Cry1Ac Increased ROS Production in D. melanogaster Midgut Tissue

Influxes of calcium ions occur when susceptible lepidopteran midgut tissue [37] or cultured cells [38] are treated with Bt toxin. Increased calcium may then accumulate in the mitochondria, causing damage [39]. RNA-seq analysis confirmed the expression of genes involved with mitochondrial production in *D. melanogaster* larvae in response to Bt toxin ingestion, which may be a response to calcium. Increased activity of mitochondria could also lead to oxidative stress following exposure to Bt insecticide via increases in ROS as a by-product of mitochondrial ATP production [40]. 

**Figure 7:**
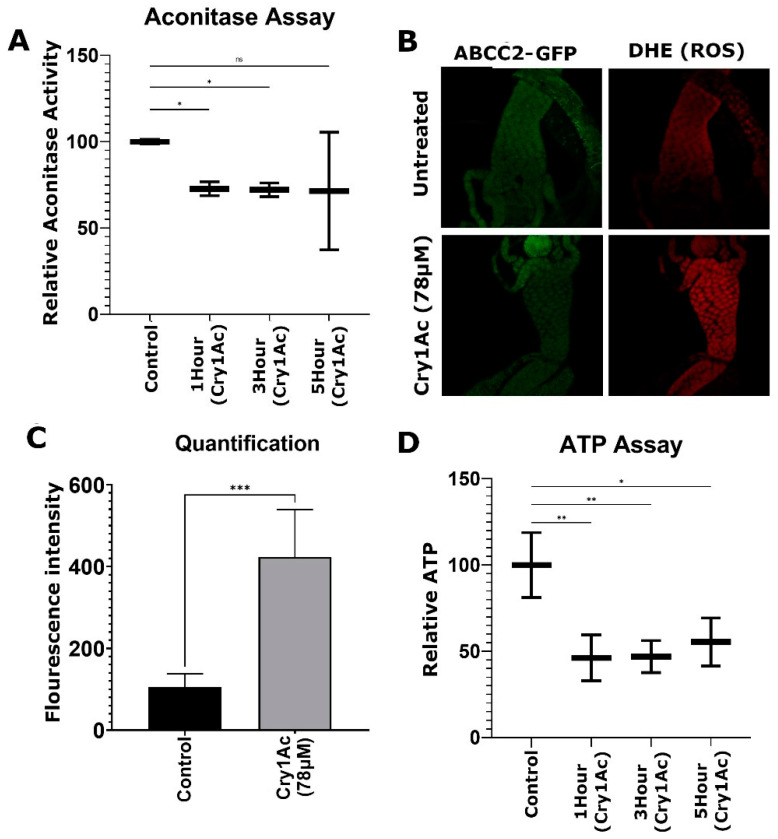
Cry1Ac causes ROS accumulation in larval midgut tissue. Larvae were exposed to 78 µM Cry1Ac for 1 h, 3 h, and 5 h. (**A**) Relative aconitase activity (n = 3 replicates of 10 larvae per treatment). (**B**) Representative images of anterior midgut stained with DHE, highlighting ROS activity following 1 h Cry1Ac larval feeding assays. (**C**) Quantification of fluorescence intensity following DHE staining (n = 7 larval midguts/treatment). (**D**) Relative ATP levels (n = 3 replicates of 10 larvae per treatment). Statistical significance was determined using one-way ANOVA, Dunnett’s multiple comparisons test (**A**,**D**), and the unpaired *t*-test (**C**), * *p* < 0.05, ** *p* < 0.01, *** *p* < 0.001.

We determined ROS activity levels and cellular ATP concentrations in transgenic third instar larval midguts that had been treated with 78 µM of Bt Cry1Ac toxin for 1 h, 3 h, or 5 h. First, mitochondrial aconitase enzyme assays were used to measure oxidative stress. Aconitase performs catalytic reactions, enabling the isomerization of citrate to isocitrate. Activity is inversely proportional to oxidative stress levels, as ROS reversibly inactivates aconitase [41]. Larvae treated with Cry1Ac had a ~23% mean reduction in levels of aconitase activity across all Cry1Ac treatment groups, which supports elevated ROS levels (Figure 7A). Next, midgut tissue was dissected from larvae treated with Cry1Ac for 1 h and analyzed with dihydroethidium (DHE) fluorescence assays to detect ROS levels. There was a measurable fourfold increase in ROS activity (Figure 7B,C).

ATP assays were then used to directly measure ATP levels in larval midgut tissue, where low levels indicated impaired mitochondrial activity due to oxidative stress. A total of 3 different treatment groups showed ATP levels to be reduced by ~55% after 1 h and 3 h and by 45% after 5 h (Figure 7D). Collectively, these data support that oxidative stress occurred via increased ROS levels when *D. melanogaster* larvae were treated with a moderate dose of Cry1Ac. 

## 3. Discussion

The morphological and genetic impacts of Cry1Ac toxin were assessed on transgenic *D. melanogaster* larva expressing a known lepidopteran Bt receptor. Feeding assays were performed over specific timeframes using moderate concentrations of the toxin mixed into the larval diet to understand how midgut cells respond to damage from insecticidal proteins. *Drosophila melanogaster* are not targets of Bt toxins and are not considered pests; however, they are one of the most intensively studied model organisms for the investigation of biological and cellular processes that are common to higher eukaryotes [42]. Primarily, we aimed to determine whether midgut cells would swell and lyse following the formation of Bt pores on the midgut epithelium, and whether the expression of genes involved in cell death pathways increased over time.

There is considerable evidence demonstrating that Bt toxins can cause cell lysis among specific cell lines [43,44]. Following pore formation and water influx, the increase in osmotic pressure results in the swelling and lysis of cells, as a mechanism to release excess water may be inefficient. In the *D. melanogaster* larval midgut, the epithelial cells act as barriers between two environments, the lumen and hemolymph, each with its own osmolarity [3]. Bt pores forming on the apical membrane cause an influx of ions and water into the cells from the lumen, thus increasing the pressure in the cell. Swelling of the basal labyrinth appeared to be greatest in the first hour of toxin exposure, and provides a potential mechanism to prevent cellular lysis. Water accumulation in the basal labyrinth may dissipate over time through fluid moving into the hemocyl. This response may prevent the midgut epithelial cells from experiencing an extreme influx of water and lysing at the concentration of the applied toxin. 

Bt formulations that include both crystal and spore mixtures, but not purified toxins, as used here, have been shown to delay the development of wild-type *D. melanogaster* [45]. *Drosophila* are highly susceptible to some pore-forming toxins, including monalysin, which is produced by the entomopathogen *Pseudomonas entomophila* [46,47]. Monalysin causes rapid disruption of enterocytes and leads to disruption and death of midgut epithelial cells, which kills both larvae and flies following infection [48]. Another pore-forming toxin, hemolysin, does not result in enterocyte death, but causes a build-up of lipid droplets in the cells, followed by proliferation of the mitochondria near the apical membrane and disruption of the endoplasmic reticulum. This leads to extrusion of the cytoplasm into the lumen and “thinning” of the epithelium without cell lysis or cell death; the enterocyte eventually recovers and returns to its original size and shape [49]. Cry1Ac did not affect the morphology as severely as monalysin did, as the tissue integrity appeared to be maintained without extensive damage. Furthermore, the cells did not appear to extrude large areas of the cytoplasm as the response to hemolysin did, but the microvilli were severely shortened, which may represent an attempt to shed areas of the plasma membrane where pore had formed. However, the morphological observations in our experiments with *Drosophila* may be due to the dose of Cry1Ac applied and the short exposure time. Higher doses of the Bt toxin are expected to increase toxicity and produce more severe phenotypes in *D. melanogaster* larvae.

Gene expression analyses have been performed on a range of insects following exposure to purified toxins, protoxins, and Bt formulations. *Tenebrio molitor* larvae treated with Cry3Aa protoxin for 24 h showed a general decrease in the expression of genes involved with digestion, although this may be caused by reduced feeding [50]. GO analysis did not identify digestion (GO:0007586) as a significant factor in *Drosophila*, which may be due to the differences in the diet and feeding behavior of these two species, the duration of exposure to Bt toxin, or the toxin class. Responses to starvation (GO:0009267) were identified in *Drosophila*. 

Immune responses to Bt in the insect gut vary among susceptible insects. When *P. xylostella* was exposed to LC_50_ concentrations of *B. thuringiensis HD-73*, a reduction in the expression of AMPs and lysozymes was reported [51]. *Trichoplusia ni* exposed to protoxin or to activated Cry1Aa, Cry1Ab, or Cry1Ac toxins showed increases in AMP expression; however, lysozyme expression was unaffected [52]. *Busseola fusca* which fed on Bt- Cry1Ab maize also showed an increase in the expression of AMP genes [53]. Sparks et al. [54] treated *Lymantria dispar* with Bt kurstaki for 24 h and concluded that immune-related genes may be directly or indirectly impacted by the bacterial formula. 

The typical Drosophila diet consists of decaying/rotting plant material; as such, insects of this species have robust immune systems with regulated signal transduction pathways, including Toll, IMD, JNK, and JAK-STAT [55]. An important function of these pathways involves the amplification of the response signal and the induction of antimicrobial activity. In this study, GO enrichment analysis did not show any consistent change in the expression of genes involved in these pathways. Similar results were generated from another *Drosophila* system, expressing the *Bombyx mori* Bt receptor *ABCC2* in third instar larval wing discs [56]. Treating cultured wing discs with activated Cry1Aa toxin caused necrotic cell death independently of apoptosis, JNK activation, or autophagy. The RNAseq results reported herein also did not implicate these cellular responses to Cry1Ac in the larval midgut tissue. 

Stress responses of mitogen-activated protein kinase (MAPK) p38 have previously been shown to play a role in Bt toxicity in *Manduca sexta* (Cry1Ab) and *Aedes aegypti* (Cry11Aa) [57]. Silencing MAPK p38 using RNAi increased toxicity in both species, suggesting that the pathway plays a role in Cry toxin defense. *Drosophila* has three MAPK p38 genes, and we observed upregulation of *p38c* (FBgn0267339) after larvae were treated on Cry1Ac for 5 h. The *Drosophila* gene’s MAPK p38c expression in the midgut is involved with oxidative stress resistance caused by ROS production, as well as starvation resistance, which is consistent with our data [58].

Specific genes involved in response to gram-negative bacteria and *Bacillus* species also have reduced expression, particularly *lysozyme X* [59]. A similar reduction in antimicrobial peptides has been seen in *D. melanogaster* in response to other pathogens and parasites [60]. Reduced expression of lysozymes may be an indirect consequence of the Cry1Ac toxin that could potentially enhance the proliferation of bacteria.

Suppression of the host immune response following Bt toxin exposure is likely to promote bacterial proliferation and lead to septicemia. The presence and abundance of bacteria in the insect midgut can affect the response to toxin exposure. Broderick et al. [61] argued that bacteria in the midgut were required for the Bt toxin activity. Subsequent evidence showed that the midgut microbiota is not required for *Bacillus thuringiensis* to have pathogenicity in *P. xylostella* or *M. sexta* [62,63]. Recent studies [64,65] have shown that toxin exposure does affect gut bacterial proliferation and diversity; however, low initial bacterial abundance negatively affects the toxin’s efficacy. TEM imaging of *D. melanogaster* midgut tissue did not show evidence of bacterial proliferation, as anti-microbial agent tegosept was used to prepare an artificial larval diet. The toxin was still active and caused clear morphological and genetic responses without requiring bacteria. The presence of bacteria in the diets and guts of *D. melanogaster* could potentially increase toxicity, as immune response genes were reduced, creating an environment for bacterial proliferation. 

The cell integrity was affected by toxin exposure, with the damage mainly focused on the apical membrane. Mitochondrial proliferation and increased vacuolization are conserved responses to toxin stress which have been described previously [66,67]. GO and KEGG analysis highlighted the increased expression of genes related to mitochondria and the tricarboxylic acid (TCA) cycle, as well as oxidative phosphorylation. Increasing the cytosolic levels of ions such as Ca^2+^ has been shown to stimulate the TCA cycle and oxidative phosphorylation processes [68]; consequently, mitochondria could be driven to work faster and consume more oxygen. Furthermore, mitochondria play a central role in the response to infection [69] by supplying energy in the form of ATP. In this case, ion influx via Bt pores may stimulate active transporters (V-ATPases, Na^+^/K^+^-ATPases, etc.) that would attempt to compensate for the sudden influx of ions caused by the formation of Bt pores generating demand for ATP. Increased mitochondrion activity generates ROS [70], which can damage DNA, proteins, and lipids. Martelli et al. [40] showed similar results in *D. melanogaster* larvae exposed to low doses of imidacloprid, a neonicotinoid insecticide. Binding of the insecticide to nicotinic acetylcholine receptors results in an influx of Ca^2+^, and ROS build up in the anterior midguts due to increased mitochondrion activity. 

This study demonstrates that activated Cry1Ac toxin directly affects the interity of *Drosophila* larval midgut tissue, expressing a lepidopteran Bt receptor protein, and induce variable transcriptomic responses from antimicrobial peptides, lysosomal genes, and the IMD pathway. Midgut cell death or activation of cell death pathways were not observed at the concentration of the toxin which was applied. Cell lysis also did not occur, and swelling of the basal labyrinth may have mitigated water influx. These results also indicate that reactive oxygen species and oxidative stress play important roles in response to Bt when insects are exposed to moderate toxin concentrations. 

## 4. Materials and Methods

### 4.1. Insect Strains and Rearing

*Drosophila melanogaster* flies were maintained on a standard media of 300 g yeast, 420 g coarse semolina, 45 g J-grade agar, 476 g molasses, 46 mL acid mix (44% propionic acid, 4.4% orthophosphoric acid), and 87.3 mL tegosept (10% methyl p-hydroxy benzoate), reaching 10 L with the addition of water. Larvae were collected after flies were allowed to lay eggs on apple juice agar plates (10 g sucrose, 250 mL apple juice, 9 g agar, 25 mL tegosept, reaching to 500 mL with the addition of water), with reconstituted yeast (~5 g of yeast in 10 mL of 10% sucrose water) at the center. Stocks were reared at 25 °C on a 24 h cycle with 14 h of light.

The NP1-GAL4 driver line was provided by Dr. Donna Denton (University of South Australia), and UAS-PxABCC2-GFP was described previously [29]. Briefly, the UAS-PxABCC2-GFP contains the *P. xylostella* ABCC2 gene (4047 bp) fused to GFP (720 bp) via a short 60 bp spacer sequence (amino acids EAAAREAAAREAAAREAAAR), as described by Arai et al. [71]. NP1-GAL4 drives UAS-linked transgene expression in the larval midgut and salivary glands.

### 4.2. Cry1Ac Production and Purification

Cry1Ac toxin was purified from *Bacillus thuringiensis* strain HD73. A single colony was grown overnight at 30 °C in 4 mL of LB, with shaking at 230 RPM. A sample of 3 mL was then used to inoculate 250 mL of LB and grown overnight at 30 °C at 200 RPM. This 250 mL culture was then inoculated with 4 × 500 mL of LB and cultured for 48 h. Cells were harvested at 8000 RPM for 8 min. The pellet was resuspended in 1 M NaCl and centrifuged at 8000 RPM for 8 min; this was repeated twice more, and then it was washed three times with water. Crystals were dissolved in lysate (50 mM Na2CO3, 50 mM EDTA, 100 mM NaCl, 3% beta-mercaptoethanol, pH to 9.6) and insoluble debris was pelleted at 8000 RPM. Cry1Ac protoxin was activated with trypsin (final concentration: 5 ng/mL) overnight at 37 °C. Protein was visualized using SDS-PAGE quantified using a Qubit 2.0 protein assay. 

### 4.3. Diet Preparation

The Cry1Ac toxin was solubilized in 50 mM Na_2_CO_3_ and thoroughly mixed into molten (50 °C) Drosophila media for a final concentration of 78 μM. The Na_2_CO_3_ concentration was normalized across all vials, and 5 mL of media aliquot was pipetted into the vials and set overnight at room temperature.

### 4.4. Midgut Dissection

Crosses were performed using homozygous *D. melanogaster* lines NP1-GAL4 (150 ♀) and UAS-PxABCC2-GFP (50 ♂), and were then allowed to lay on apple juice agar plates in cages for 2 h. Plates were maintained at 25 °C for four days; then, the resulting third instar larvae were transferred to the diet with activated Cry1Ac toxin (78 μM) for 1, 3, or 5 h, or to the toxin-free control diet for 1 h. Midguts from the larvae were dissected in phosphate-buffered saline (PBS); then, gastric caeca and short sections of the attached midgut were collected. Midgut samples were frozen on dry ice for subsequent RNA isolation or fixed in freshly made 4% paraformaldehyde for transmission electron microscopy.

### 4.5. RNA Extraction

Dissected midguts were pooled into groups of eight, with three replicates for each timepoint. The tissues were homogenized using 3 mm stainless steel ball-bearings in a TissueLyser II (QIAGEN Pty Ltd., Clayton, Australia) at 30 Hz for 1 min with pre-chilled support blocks. Total RNA was isolated using RNeasy Lipid Tissue Mini Kit (QIAGEN Pty Ltd., Clayton, Australia) according to the manufacturer’s instructions. Concentrations were quantified using a NanoDrop (Life Technologies Thermo Fisher Scientific Aust Pty Ltd., Scoresby, Australia).

### 4.6. Transmission Electron Microscopy

After collecting midguts in 4% paraformaldehyde, samples were postfixed in 2% osmium tetroxide (OsO_4_), suspended in PBS for 1 h, and then dehydrated through a series of ethanol and propylene oxide washes on a rotator. Tissue samples were embedded in resin and polymerized in an oven at 70 °C for 24 h. Sections were cut from the resin blocks with a Leica EM UC6 Ultra Microtome at 70 nm, placed on formvar/carbon grids (Sigma–Aldrich, Sydney, Australia ), and stained with 4% uranyl acetate (10 min) followed by Reynold’s lead citrate (10 min) before being viewed on a Tecnai G2 Spirit transmission electron microscope (FEI) at 100 kv.

### 4.7. ImageJ Quantification

Quantification of the lengths of the microvilli, the thicknesses of the peritrophic membranes, and the vacuole area were determined from TEM images using ImageJ software [72]. Three independent biological replicates were analyzed for each treatment. The lengths of the microvilli and the thicknesses of the peritrophic membranes were determined by taking the average of >10 measurements per image from ~14 images. The lengths and thicknesses, in µm, were calculated using image scale bars. The volumes of the vacuoles was determined by taking the average radius of >10 vacuoles per image. The radius measurements were then converted to µm using image scale bars, and the volume was calculated according to the following formula.

$$V=\frac{4}{3}\pi r^{3}$$

### 4.8. RNA-Seq Library Preparation and Sequencing

RNA-seq library preparation and sequencing were performed at the Adelaide Cancer Research Foundation (ACRF). The quality of the total RNA was evaluated using an Agilent 2100 Bioanalyzer. Poly-A purification of RNA was performed using the NEBNext^®^ Poly(A) mRNA Magnetic Isolation Module, and library construction was completed with the KAPA Stranded RNA-Seq Library Preparation Kit. Sequencing was carried out on a HiSeq 2500 (Illumina) in 75 bp, single-end sequencing reactions. 

### 4.9. Sequence Read Alignment

Adapter and low-quality bases (Phred score < 20) were trimmed using Trimmomatic (v0.38). Trimmed reads were mapped to the *D. melanogaster* genome (BDGP6), downloaded from Ensembl (ftp.ensembl.org), or the *P. xylostella* genome (GCA_019096205.1), using STAR (v2.5.3a) [73]. Transcript counts were produced using Feature Counts (v1.5.2) [74].

### 4.10. Differential Gene Expression Analysis

Differential expression analyses were carried out for each insecticide treatment group versus the untreated control group using Bioconductor package edgeR (3.24.3) [75] with Genewise Negative Binomial Generalized Linear Models and Quasi-likelihood tests. Genes with >1 counts per million reads in all replicate samples were considered abundant, and the remainders were removed. A false discovery rate (FDR) of less than 0.05 was used as the threshold p-value to judge the significance of gene expression differences. Genes were considered differently expressed with an FDR ≤ 0.05 and greater than two-fold change (absolute value of the log_2_ ratio > 1.2).

### 4.11. Gene Ontology (GO) and Kyoto Encyclopedia of Genes and Genomes (KEGG) Analysis

The bioconductor package limma (v3.38.3) [76] was used to carry out the gene enrichment analyses. The goana and kegga functions were used to map genes to terms in the GO database and pathways in the KEGG database. The functions topGO and topKEGG were used to extract the top GO terms and KEGG pathways, respectively. An FDR < 0.05 and a change greater than two-fold were used as the thresholds for the genes used in the analysis. 

### 4.12. Analysis of Gene Co-Expression

Significantly differentially expressed genes were used for co-expression analysis with CLUST version 1.12.0 [35]. GO analysis was carried out on the list of genes from each cluster using the Database for Annotation, Visualization, and Integrated Discovery (DAVID, version 6.8) [36], and a *p* < 0.05 was considered statistically significant.

### 4.13. cDNA Synthesis and qPCR

Complementary DNA (cDNA) was synthesized from total RNA using SuperScript IV reverse transcriptase (Thermo Fisher Scientific Aust Pty Ltd., Scoresby, Australia) according to the manufactures protocol. Total RNA, 10 mM dNTPs, and oligod(T)20 primer (50 µM) were heated to 65 °C for 5 min; then, 5-times SuperScript IV reaction buffer, 100 mM DTT, RNase OUT, and SuperScript IV reverse transcriptase were added, and the reaction was incubated at 23 °C for 10 min. The cDNA was synthesized at 50 °C for 10 min, then inactivated at 80 °C for 10 min. A 1:1 dilution of the synthesized cDNA was made with purified water. The relative expression (fold change) of each gene compared to the expression level control was calculated using the 2^−△△Ct^ method. Quantitative PCR (qPCR) was carried out using the One Step RT-PCR kit SensiFAST SYBR (Bioline, Eveleigh, Australia) according to the manufacturer’s instructions on a StepOnePlus (Applied Biosystems Thermo Fisher Scientific Aust Pty Ltd., Scoresby, Australia) cycler (Table S8).

### 4.14. ROS Staining

The ROS staining protocol was adapted from Owusu-Ansah et al. (2008). Third instar larvae were fed on a standard diet containing 78 µM of activated Cry1Ac toxin for 1 h. Control larvae were fed on a toxin free diet. Larval midguts were then dissected in Schneider’s Drosophila Media 1x (GIBCO) and incubated in 1 mL of the same media containing 30 μM DHE (Thermo Fisher Scientific) for ~7 min in the dark on an orbital shaker. The midgut tissue was washed three times (5 min each) with Schneider’s media, then fixed in 10% paraformaldehyde for 5 min at room temperature and, finally, rinsed with PBS and mounted in Fluoro-mount (Sigma). Confocal microscopy images were obtained using a Confocal Olympus FV3000 Microscope at 200x magnification (excitation/emission: 518/605 nm).

### 4.15. ATP and Aconitase Activity Assays

Third instar larvae were fed an activated Cry1Ac (78 µM) diet for 1 h, 3 h, or 5 h, and control larvae were fed a standard diet. Larval ATP levels were quantified using the ATP assay kit (Abcam) according to manufacturer’s instructions. Each condition was tested in triplicate, with 10 larvae per sample. Fluorescence was measured at 534/587 nm using a Synergy™ HTX Multi-Mode Microplate Reader. Aconitase activity was measured using the Aconitase Activity Assay Kit (Sigma), according to the manufacturer’s instructions. Each condition was tested in triplicate, with 10 larvae per sample. Absorbance was measured at 450 nm using the Synergy™ HTX Multi-Mode Microplate Reader.

### 4.16. Statistical Analysis and Imaging

Graphs were made using R (v.3.24.3) or GraphPad Prism (GraphPad Software, San Diego, CA, USA, v9.0.0). Figures were designed using the Open−Source Image Editor GIMP (v2.10.22). Statistical significance was determined by means of one-way ANOVA, Dunnett’s multiple comparisons test, or the unpaired t-test using GraphPad Prism (GraphPad Software, San Diego, CA, USA, v9.0.0), and *p*-values of * *p* < 0.05, ** *p* < 0.01, *** *p* < 0.001 were considered significant.

## Figures and Tables

**Table 1:** List of essential genes involved in immune response to bacterial infection IMD, lysozymes, and AMP (“∨”, downregulated; “∧”, upregulated; logFC > 1.2; FDR < 0.05; “ns”, not significant; logFC < 1.2; and FDR > 0.05).

				1 H			3 H			5 H	
	FlyBase ID Symbol			logFC	FDR		logFC	FDR		logFC	FDR
**IMD**	FBgn0038928	Fadd	ns	−0.049	1.000	ns	0.174	1.000	∧	1.433	0.004
	FBgn0014018	Rel	∧	2.377	0.001	∧	1.208	0.048	ns	0.703	0.443
	FBgn0010303	hep	ns	0.029	1.000	ns	1.596	0.033	ns	0.959	0.240
	FBgn0001297	kay	∧	2.154	0.001	ns	0.959	0.121	ns	0.519	0.766
**Lys**	FBgn0004427	LysD	ns	−0.689	0.654	ns	−1.311	0.208	∨	−1.901	0.046
	FBgn0004431	LysX	∨	−7.066	0.000	∨	−6.186	0.000	∨	−3.052	0.000
**AMP**	FBgn0038530	AttD	∧	3.649	0.004	∧	4.053	0.002	∧	5.054	0.001
	FBgn0000276	CecA1	∧	2.722	0.005	∧	2.471	0.008	∧	1.731	0.049
	FBgn0000277	CecA2	∧	3.337	0.003	∧	2.933	0.004	∧	2.419	0.012
	FBgn0004240	DptA	∨	−3.313	0.003	∨	−2.084	0.027	∨	−2.056	0.025
	FBgn0041581	AttB	∧	3.728	0.004	∧	4.030	0.002	ns	2.078	0.055
	FBgn0262881	CG43236	∨	−3.635	0.020	∨	−2.906	0.050	ns	−1.919	0.160

**Table 2:** List of essential genes involved in autophagy and apoptosis (see Table 1 for description).

	Flybase ID Symbol			1 H			3 H			5 H	
				logFC	FDR		logFC	FDR		logFC	FDR
Autophagy	FBgn0026404	Dronc	∧	2.416	0.004	∧	1.643	0.036	∧	1.832	0.017
	FBgn0010501	Dcp-1	ns	0.827	0.153	∧	1.344	0.004	∧	2.427	0.000
	FBgn0025624	CG4025	ns	0.848	0.237	ns	0.699	0.508	∧	1.629	0.006
	FBgn0265464	Traf6	ns	0.105	1.000	ns	0.773	0.211	∧	1.204	0.005
	FBgn0010213	Sod2	∧	1.426	0.003	ns	0.987	0.040	ns	0.242	1.000
	FBgn0010303	hep	ns	0.029	1.000	∧	1.596	0.033	ns	0.959	0.240
	FBgn0002567	Rab32	ns	−0.018	1.000	∨	−1.454	0.005	ns	−0.363	1.000
	FBgn0034897	Sesn	ns	−0.583	0.651	∨	−2.211	0.002	ns	−1.124	0.077
	FBgn0261108	Atg13	∨	−1.495	0.010	ns	−0.404	1.000	ns	0.040	1.000
	FBgn0003997	hid	∨	−4.011	0.004	ns	−2.077	0.052	ns	0.022	1.000
	FBgn0262656	Myc	∨	−3.611	0.003	ns	−1.549	0.142	ns	−1.511	0.137
	FBgn0039044	p53	∨	−1.857	0.011	ns	−1.097	0.145	ns	−0.429	0.875
	FBgn0086357	Sec61alpha	∧	1.271	0.022	ns	0.176	1.000	ns	−0.231	1.000
	FBgn0010638	Sec61beta	∧	1.256	0.019	ns	0.490	0.907	ns	−0.447	0.893
	FBgn0003360	sesB	∧	1.499	0.004	ns	0.827	0.206	ns	−0.219	1.000
Apoptosis	FBgn0026404	Dronc	∧	2.416	0.004	∧	1.643	0.036	∧	1.832	0.017
	FBgn0067102	GlcT	∧	2.137	0.001	ns	1.140	0.016	∧	1.247	0.007
	FBgn0003892	ptc	∨	−1.535	0.009	ns	−1.166	0.046	∨	−1.472	0.010
	FBgn0010501	Dcp−1	ns	0.827	0.153	∧	1.344	0.004	∧	2.427	0.000
	FBgn0015245	Hsp60A	ns	0.322	1.000	∨	−1.464	0.007	∨	−1.620	0.004
	FBgn0033784	SCCRO3	ns	0.457	0.912	ns	1.072	0.028	∧	1.534	0.002
	FBgn0022027	Vps25	ns	0.335	1.000	ns	1.072	0.020	∧	1.345	0.003
	FBgn0025878	wrapper	ns	−0.884	0.608	ns	3.667	0.002	∧	2.580	0.012
	FBgn0013762	Cdk5	ns	0.791	0.316	ns	0.877	0.236	∧	1.246	0.034
	FBgn0033783	CG17019	ns	0.686	0.506	ns	1.049	0.147	∧	1.679	0.012
	FBgn0053346	CG33346	ns	−1.041	0.273	ns	−0.255	1.000	∧	3.068	0.002
	FBgn0036831	CG6839	ns	−0.417	0.911	ns	−1.251	0.183	∨	−2.384	0.009
	FBgn0036165	chrb	ns	0.291	1.000	ns	0.540	0.801	∧	1.675	0.011
	FBgn0038928	Fadd	ns	−0.049	1.000	ns	0.174	1.000	∧	1.433	0.004
	FBgn0259108	futsch	ns	−1.558	0.114	ns	−1.085	0.362	∨	−1.922	0.048
	FBgn0013726	pnut	ns	0.478	0.881	ns	0.941	0.075	∧	1.361	0.004
	FBgn0034279	CG18635	ns	1.028	0.142	∧	1.407	0.032	ns	0.913	0.219
	FBgn0045035	tefu	ns	−0.949	0.222	∨	−1.619	0.021	ns	−1.068	0.132
	FBgn0004569	aos	∨	−1.380	0.014	ns	−0.623	0.636	ns	−0.724	0.370
	FBgn0261108	Atg13	∨	−1.495	0.010	ns	−0.404	1.000	ns	0.040	1.000
	FBgn0264291	Det	∨	−1.847	0.030	ns	−1.130	0.216	ns	−0.875	0.372
	FBgn0024732	Drep1	∨	−1.382	0.047	ns	−1.366	0.053	ns	−0.824	0.335
	FBgn0003997	hid	∨	−4.011	0.004	ns	−2.077	0.052	ns	0.022	1.000
	FBgn0262656	Myc	∨	−3.611	0.003	ns	−1.549	0.142	ns	−1.511	0.137
	FBgn0039044	p53	∨	−1.857	0.011	ns	−1.097	0.145	ns	−0.429	0.875
	FBgn0038519	Prx3	∧	1.442	0.005	ns	0.944	0.090	ns	−0.452	0.911
	FBgn0003360	sesB	∧	1.499	0.004	ns	0.827	0.206	ns	−0.219	1.000

## Data Availability

RNA-seq datasets are available through the NCBI BioProject PRJNA871301.

## Supplementary Materials

The following supporting information can be downloaded at: https://www.mdpi.com/article/10.3390/toxins15050323/s1; Table S1: RNA-seq library statistics, showing total quality-filtered reads, total reads mapped; Table S2: Significant GO terms identified from transcriptomic analysis of *D. melanogaster* larval midgut tissue treated with Cry1Ac toxin; Table S3: Top KEGG enrichment terms for *D. melanogaster* midgut transcriptome analysis (Cry1Ac treated versus control); Table S4: List of terms identified using DAVID GO that are significantly enriched within a gene cluster, generated using the Clust program (Figure 4); Table S5: Genes grouped into clusters as shown in Figure 4; Table S6: Total list of differentially expressed genes (8132 genes); Table S7: List of common differentially expressed genes between all treatments (214 genes); Table S8: Quantitative PCR primer sequences.
